# Factors Associated With Postoperative Pain Severity and Patient Satisfaction Among Post‐Caesarean Women in Rajshahi District, Bangladesh

**DOI:** 10.1155/anrp/1056316

**Published:** 2026-09-18

**Authors:** Farhana Hasan, Abu Sayed Md. Al Mamun, Mst Sabina Yeasmin Swapna, Md. Kaderi Kibria, Masuma Amanullah, Md. Golam Hossain

**Affiliations:** ^1^ Health Research Group, Department of Statistics, University of Rajshahi, Rajshahi 6205, Bangladesh, ru.ac.bd; ^2^ Shaheed Syed Nazrul Islam Medical College, Kishoreganj, Bangladesh

**Keywords:** APS-POQ-R, Bangladesh, caesarean delivery, pain management, pain severity, patient satisfaction, postoperative pain

## Abstract

**Background:**

Caesarean delivery (CD) is a common surgical procedure, and effective postoperative pain (POP) management is essential for maternal recovery and psychological well‐being. Despite the increasing use of CD in Bangladesh, evidence regarding POP severity, pain‐related outcomes, and patient satisfaction remains limited, particularly in regional healthcare settings. This study assessed POP severity, pain management outcomes, patient satisfaction, and factors associated with satisfaction among women undergoing CD in Rajshahi District, Bangladesh.

**Methods:**

A hospital‐based cross‐sectional study was conducted between November and December 2024 among 297 women who had undergone CD within the previous 24 h in selected public and private healthcare facilities in Rajshahi District. Data were collected through face‐to‐face interviews using the Bangla version of the Revised American Pain Society Patient Outcome Questionnaire (APS‐POQ‐R). Descriptive statistics, chi‐square tests, and binary logistic regression analyses were performed to identify factors associated with satisfaction with pain management.

**Results:**

Substantial POP was reported during the first 24 h after CD. Overall, 84.2% of participants experienced moderate‐to‐severe pain, while 72.1% rated their worst pain as severe. Pain substantially interfered with physical activities, with moderate‐to‐severe interference reported for activities in bed (84.9%) and out of bed (76.1%). Emotional distress was also considerable, particularly fear (78.1%), anxiety (68.4%), helplessness (54.9%), and depression (52.9%). Despite this pain burden, 65.0% of women were satisfied with their pain management. However, 35.0% reported no pain relief, while only 5.7% reported complete relief. Adverse drug effects were reported by 34.0% of participants. In multivariable analysis, women aged 25–29 years had lower odds of satisfaction than those aged ≥ 30 years (AOR = 0.43, 95% CI: 0.19–0.96), whereas overweight women had higher odds of satisfaction than obese women (AOR = 3.27, 95% CI: 1.26–8.49). Those who received no pain medication also had higher odds of satisfaction (AOR = 5.49, 95% CI: 1.37–22.06).

**Conclusion:**

Women undergoing CD in Rajshahi District experienced a substantial burden of POP, physical interference, and emotional distress despite relatively high overall satisfaction with pain management. Limited pain relief and inadequate pharmacological management appear to be important concerns. Strengthening standardized, multimodal, and patient‐centered POP management may improve pain control and maternal recovery following CD.

## 1. Introduction

Caesarean delivery (CD) is one of the most frequently performed surgical procedures worldwide and plays a vital role in preventing maternal and neonatal morbidity and mortality when vaginal delivery presents significant health risks [1]. Over the past 2 decades, the global use of CD has increased substantially, with particularly rapid growth observed in low‐ and middle‐income countries (LMICs) [2–5]. Bangladesh has experienced a remarkable rise in CD utilization, exceeding the rate recommended by the World Health Organization (WHO), which suggests that population‐level CD rates above 10%–15% are unlikely to yield additional maternal or neonatal health benefits [6]. While medically indicated CDs can be life‐saving, the growing prevalence of surgical births has heightened concerns regarding the quality of postoperative maternal care, particularly pain management during the immediate recovery period [7–10].

Postoperative pain (POP) following CD remains one of the most common and challenging complications affecting maternal recovery [11]. Inadequately managed pain may delay ambulation, impair physical functioning, hinder breastfeeding initiation, disrupt maternal–infant bonding, prolong hospital stays, and increase the risk of postpartum psychological distress [12]. Maternal body weight may also influence POP outcomes; higher body mass index (BMI) has been associated with altered pharmacokinetics of analgesic medications, including changes in drug distribution and clearance, which may result in inadequate dosing and suboptimal pain relief among women with higher BMI undergoing surgery [13]. Previous studies have further demonstrated that severe acute POP is associated with persistent pain, reduced quality of life, and postpartum depression [14]. Consequently, effective pain management has become a fundamental component of quality obstetric care and an important indicator of patient‐centered healthcare delivery. Current evidence‐based recommendations advocate a multimodal approach to POP management, including the use of nonopioid analgesics, opioid medications when appropriate, regional anesthesia techniques, and enhanced recovery after surgery (ERAS) protocols [15–17]. These approaches have been shown to improve pain control, facilitate early recovery, and enhance patient satisfaction [18, 19]. However, the implementation of such strategies remains inconsistent across many LMIC settings. In Bangladesh, variations in healthcare resources, provider practices, medication availability, and institutional protocols may contribute to differences in POP experiences among women undergoing CD [20–22]. Furthermore, disparities between public and private healthcare facilities may influence the quality and effectiveness of pain management services [23, 24]. The increasing acceptance of CD in Bangladesh has been attributed to several factors, including greater access to institutional delivery services, expansion of private healthcare facilities, physician recommendations, fear of labor pain, perceived safety advantages of surgical delivery, and changing maternal preferences among urban and educated women [3, 25]. Despite these developments, concerns regarding postoperative recovery remain important because many women continue to experience considerable pain following surgery. Cultural norms in South Asian societies may further influence pain reporting, as women often perceive postoperative discomfort as an expected consequence of childbirth and may be reluctant to communicate their pain experiences to healthcare providers [26, 27]. Such factors may contribute to the underrecognition and undertreatment of POP. Patient satisfaction has emerged as a key outcome measure for evaluating healthcare quality and the effectiveness of pain management interventions [28]. Satisfaction reflects not only the degree of pain relief achieved but also patients’ perceptions of communication, responsiveness, emotional support, and overall quality of care [28]. Several studies have examined POP and pain management outcomes following CD [7, 11, 29–31]; however, most investigations have focused primarily on clinical outcomes, analgesic effectiveness, or surgical indicators. Comparatively fewer studies have simultaneously assessed both POP severity and patient satisfaction, particularly in Bangladesh. Moreover, evidence from northwestern Bangladesh, including the Rajshahi District, remains limited. Understanding both the severity of pain and women’s perceptions of pain management is essential for developing patient‐centered strategies that improve postoperative recovery and maternal well‐being.

The present study was guided by Donabedian’s Structure–Process–Outcome model of healthcare quality. According to this framework, healthcare outcomes are influenced by structural factors (e.g., healthcare facilities, availability of resources, and institutional capacity) and process factors (e.g., pain assessment, administration of analgesics, and patient–provider communication). In the context of post‐caesarean care, sociodemographic characteristics, clinical conditions, and pain management practices may collectively influence POP experiences and subsequent satisfaction with care. Applying this framework provides a conceptual basis for examining the determinants of pain management outcomes among women undergoing CD. Therefore, this study aimed to assess POP severity and patient satisfaction among women who underwent CD in Rajshahi District, Bangladesh. Specifically, the study sought to (i) evaluate the severity and multidimensional impact of POP during the first 24 h following CD; (ii) assess women’s satisfaction with POP management; and (iii) identify sociodemographic, clinical, and treatment‐related factors associated with satisfaction with pain management. The findings are expected to inform the development of standardized, evidence‐based, and patient‐centered pain management strategies to improve maternal recovery and quality of care in Bangladesh.

## 2. Methods

### 2.1. Study Design and Data Source

A hospital‐based cross‐sectional study was conducted between 22 November and 30 December 2024 in Rajshahi District, northwestern Bangladesh. Rajshahi serves as a major administrative and healthcare center and contains a mix of government and private healthcare facilities providing comprehensive obstetric services. To ensure representation across diverse healthcare settings, the study was conducted across seven selected healthcare facilities, comprising one government hospital, three private hospitals, and three specialized clinics performing CD. Across the selected government hospital, private hospitals, and specialized clinics, POP management commonly involved pharmacological analgesia, primarily nonopioid analgesics (e.g., paracetamol and NSAIDs), with opioid analgesics such as pethidine used selectively for more severe pain. Antibiotics were also routinely co‐administered as part of standard postoperative care. Analgesic prescribing and administration practices were determined by attending clinicians on a case‐by‐case basis, as no uniform, facility‐wide standardized pain management protocol was documented across the study sites.

### 2.2. Study Population and Eligibility Criteria

The study population consisted of women aged 15–49 years who had undergone CD and were admitted to the selected healthcare facilities during the study period. Women were eligible to participate if they had undergone CD within the preceding 24 h, were clinically stable, and were able to communicate and provide informed consent. Women with severe postoperative complications, cognitive impairment, or critical illness preventing participation in an interview were excluded.

### 2.3. Sample Size and Sampling Technique

The sample size was determined using a single population proportion formula [32], where *n* represents the required sample size, *Z* = 1.96 for a 95% confidence level, *p* = 0.76, and *d* = 0.05.

The expected proportion (76%) was derived from previous studies reporting approximately three‐quarters of post‐caesarean mothers being satisfied with pain management services [30, 33]. Using these assumptions, the minimum required sample size was estimated at 280 participants. To improve statistical precision and account for possible incomplete responses, the final sample was increased to 297 women, who were ultimately enrolled in the study. A two‐stage sampling approach was employed. Facilities were considered eligible for inclusion if they were located within Rajshahi District, routinely performed CDs, and had a functioning postoperative maternity/inpatient ward permitting patient interviews within 24 h of surgery. In the first stage, one government hospital, three private hospitals, and three specialized clinics were selected using simple random sampling from this list of eligible facilities providing CD services in Rajshahi District. In the second stage, eligible women who had undergone CD within the previous 24 h were consecutively recruited from the selected facilities until the required sample size was achieved. The final sample comprised 93 participants from the government hospital, 144 from private hospitals, and 60 from clinics (Table S1).

### 2.4. Data Collection Procedure

Data were collected through face‐to‐face interviews using a structured questionnaire administered by trained data collectors. Interviews were conducted once for each participant within the first 24 postoperative hours following CD. The questionnaire consisted of two sections. The first section collected information on sociodemographic, anthropometric (height and weight, used to calculate BMI), obstetric, and clinical characteristics. The second section comprised the Bangla‐translated version of the Revised American Pain Society Patient Outcome Questionnaire (APS‐POQ‐R) [34]. Prior to data collection, participants were informed about the objectives of the study and written informed consent was obtained.

### 2.5. Translation and Validation of the APS‐POQ‐R

The APS‐POQ‐R was translated into Bangla using a forward–backward translation procedure. Initially, bilingual experts translated the original English version into Bangla. A separate bilingual translator subsequently back‐translated the instrument into English to ensure semantic equivalence. The translated version was reviewed by five experts in public health and clinical care to assess content validity and cultural appropriateness. A pilot study was conducted among post‐caesarean women outside the study facilities to evaluate clarity, comprehensibility, and feasibility. Necessary modifications were incorporated before the final survey. Internal consistency reliability was assessed using Cronbach’s alpha coefficient. The overall Cronbach’s alpha value of the Bangla APS‐POQ‐R was 0.89, indicating excellent reliability [35, 36]. All corrected item–total correlation coefficients exceeded the recommended threshold of 0.30, supporting item validity [36] (Table S2).

### 2.6. Measurement of Postoperative Pain and Satisfaction

POP and treatment outcomes were assessed using the APS‐POQ‐R [34], which employs multiple response formats across its items: an 11‐point numeric rating scale (NRS), percentage‐scale items, and dichotomous (yes/no) items. Pain assessment referred to participants’ experiences during the first 24 h following CD. Participants rated their worst, least, and average pain intensity during this period using an 11‐point NRS ranging from 0 (“no pain”) to 10 (“worst possible pain”). Participants also reported the proportion of time spent in severe pain over the same 24‐h period, assessed using a percentage scale (0%–100%). In addition to pain intensity, the APS‐POQ‐R assessed the extent to which pain interfered with physical activities (in bed and out of bed) and emotional states such as anxiety, depression, fear, and helplessness, each rated using an 11‐point NRS. Two items, experience of any medication‐related side effects and receipt of information about pain‐treatment options, were assessed using a dichotomous yes/no format. The APS‐POQ‐R also assessed patients’ perceptions of the process of care. Specifically, participants reported whether they had received information about pain‐treatment options (yes/no) and the degree to which they felt involved in decisions regarding their pain treatment, rated using an 11‐point NRS. Additionally, among participants who reported receiving information, perceived helpfulness was rated on a 0–10 scale. Overall pain relief and satisfaction with pain management were assessed as two separate, conceptually distinct items. Pain relief was measured on a percentage scale (0%–100%), reflecting the extent of relief obtained from the treatment received. Satisfaction with pain management was measured independently using an 11‐point NRS (0–10). No composite score combining these two items was constructed; satisfaction was treated as the primary outcome variable and is described further in Section 2.7. For descriptive purposes, individual pain intensity scores were additionally categorized as no pain at all (0), mild (1–3), moderate (4–7), and severe (8–10), consistent with widely used pain‐severity classification thresholds [37]; this classification was applied to worst and average pain intensity scores for descriptive purposes and is not part of the original APS‐POQ‐R scoring.

### 2.7. Study Variables

**FIGURE 1:**
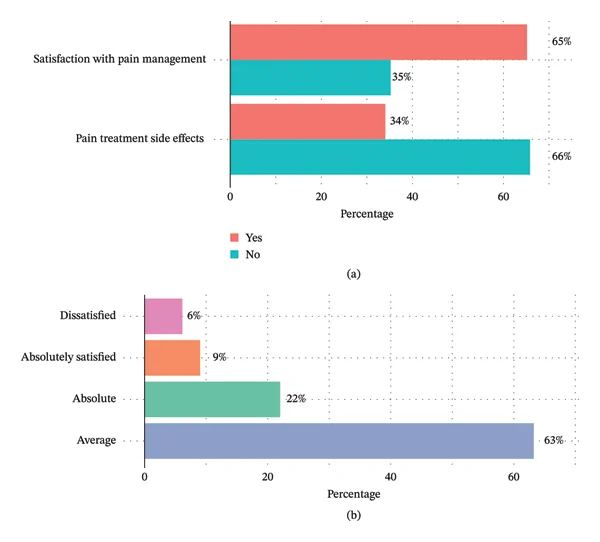
Distribution of patient’s satisfaction and prevalence of side effects. (a) Pain management status and pain treatment side effect status among the participants. (b) Respondent’s pain treatment satisfaction level.

The primary outcome of this study was satisfaction with pain management following CD. Because participants’ reported degree of pain relief and their satisfaction rating on the APS‐POQ‐R (Section 2.6) were closely related in this dataset, and because a substantial proportion of women reported receiving no pain relief at all, the pain relief item (percentage scale, 0%–100%, reflecting the extent of relief obtained from the treatment received) was used to operationalize the dichotomized outcome for bivariate and multivariable analysis: no relief at all (0%; code = 0, “dissatisfied”) versus any relief (> 0%; code = 1, “satisfied”) [38]. The original four‐category distribution (no relief, mild, moderate, and complete satisfaction) is presented descriptively in Figure 1b, while the dichotomized satisfied/dissatisfied outcome used in subsequent analyses is presented in Figure 1a. No composite score combining the satisfaction item and the pain relief item was created; although both constructs relate to treatment outcomes, they were measured independently, satisfaction on an 11‐point NRS and pain relief on a percentage scale, and were treated as separate variables throughout the analysis. Pain relief was reported descriptively and was not incorporated into the outcome definition. Selection of explanatory variables was informed by previous literature and the study’s conceptual framework [11]. Sociodemographic characteristics (age, education, household income, husband’s education, and occupation), anthropometric characteristics (BMI, calculated as weight in kilograms divided by height in meters squared and classified as underweight (< 18.5 kg/m^2^), normal weight (18.5–22.9 kg/m^2^), overweight (23.0–27.4 kg/m^2^), and obese (≥ 27.5 kg/m^2^) according to the WHO Asian population‐specific BMI classification [39]), obstetric characteristics (age at first birth), pain characteristics (pre‐delivery and post‐delivery pain severity), and treatment‐related factors (use and type of analgesics) were included because previous studies have demonstrated their potential influence on POP experiences and satisfaction with pain management among women undergoing CD. BMI was included as an explanatory variable because higher BMI has been associated with altered drug pharmacokinetics and potentially reduced analgesic efficacy, which may influence POP experiences and satisfaction with pain management [13]. As this study relied on patient‐reported data rather than clinical chart review, information on analgesic type and use reflects participants’ self‐reported recall of medication received from hospital staff during their hospital stay, rather than documented institutional prescribing records. Pre‐delivery pain referred to pain experienced by participants prior to undergoing CD, assessed via participant self‐report and classified as no pain, mild pain, or severe pain based on the participant’s subjective description of pain intensity [40]. The category ‘painkiller’ broadly referred to nonopioid analgesics reported by participants, without further specification of individual drug names, dosages, or administration frequency; pethidine, an opioid analgesic, was recorded as a separate category. Information on specific drug names, dosages, and administration frequency (e.g., for painkillers or pethidine) was not collected in this study, as data collection relied on patient self‐report rather than clinical chart review. The specific source of pre‐delivery pain (e.g., labor contractions, pregnancy‐related discomfort, or other causes) was not further differentiated in this study. Post‐delivery pain referred to pain experienced during the first 24 h following CD, as assessed using the APS‐POQ‐R. Information on the type of anesthesia administered during surgery (e.g., spinal, epidural, or general) and intraoperative analgesic use was not collected, as data collection focused on participants’ self‐reported pain experiences and treatment received during the first 24 h following CD. Antibiotics were included in the treatment‐related variable as they are commonly co‐administered with analgesics as part of standard postoperative care following CD for infection prophylaxis, rather than for direct pain relief; their inclusion reflects overall treatment received rather than a pain‐specific intervention.

### 2.8. Statistical Analysis

Data were cleaned, coded, and analyzed using IBM SPSS Version 22.0. Descriptive statistics, including frequency distributions and graphical representations, were used to summarize the sociodemographic profile of the participants and the prevalence of POP. Given that the APS‐POQ‐R comprises items with different response formats, continuous variables measured on the 11‐point NRS were summarized as means and standard deviations; the percentage‐scale item (proportion of time in severe pain) was summarized as a percentage; and dichotomous (yes/no) items were summarized as frequencies and percentages. The chi‐square (*χ* ^2^) test was used to find the association between categorical independent variables and pain satisfaction [41]. Multiple logistic regression model was utilized to identify significant determinants of satisfaction [42]. Model fitness was confirmed using the Hosmer–Lemeshow test [43]. The predictive accuracy was further validated using receiver operating characteristic (ROC) curves [44]. The value of *p* < 0.05 was considered as statistically significant in the analysis.

### 2.9. Ethics Approval

This study involved human participants and received ethical approval from the Ethical Review Committee of the Institute of Biological Sciences, University of Rajshahi, Bangladesh (Memo No. 192(69)/320/IAMEBBC/IBSC). Written informed consent was obtained from each selected participant prior to their inclusion in the study. All participants voluntarily agreed to participate after being fully informed about the nature and purpose of the research.

## 3. Results

### 3.1. Sociodemographic Profiles of the Respondents

A total of 297 women who had undergone CD were included in the study. As shown in Table 1, most participants resided in urban areas (73.7%) and had secondary or higher education (83.2%). Similarly, 88.2% of participants’ husbands had secondary or higher education. Most respondents were aged 19–29 years (69.7%), and 50.8% reported their first childbirth at or before 19 years of age. Regarding nutritional status, 43.1% of participants were overweight and 8.4% were obese. The most common occupations among husbands were service employment (37.7%) and business (37.4%). More than half of the households (53.2%) reported a monthly family income of BDT 25,000 or more. Detailed sociodemographic, educational, economic, and anthropometric characteristics are presented in Table 1.

**TABLE 1:** Sociodemographic and anthropometric characteristics of the respondents (*n* = 297).

Variable	Frequency (%)
*Location*	
Urban	219 (73.7)
Rural	78 (26.3)

*Respondent’s education level*	
Uneducated	6 (2.0)
Primary	44 (14.8)
Secondary	114 (38.4)
Higher	133 (44.8)

*Husband’s education level*	
Uneducated	10 (3.4)
Primary	25 (8.4)
Secondary	38 (12.8)
Higher	224 (75.4)

*Respondent’s age (in years)*	
≤ 18	42 (14.1)
19–24	105 (35.4)
25–29	102 (34.3)
≥ 30	48 (16.2)

*Age at first birth*	
≤ 19	151 (50.8)
20–24	97 (32.7)
25–29	40 (913.5)
≥ 30	9 (3.0)

*Body mass index*	
Normal weight	133 (44.8)
Overweight	128 (43.1)
Obese	25 (8.4)

*Husband’s occupation*	
Service holder	112 (37.7)
Businessman	111 (37.4)
Farmer	15 (5.1)
Daily wager	59 (19.9)

*Monthly family income (in BDT)*	
≤ 20,000	102 (34.3)
20,001–25,000	37 (12.5)
25,000+	158 (53.2)

### 3.2. Pain Experience and Management Practices

As shown in Table 2, 39.1% of participants reported that their pain had started before delivery, whereas 60.9% reported no pain before delivery. Overall, 58.6% reported no pain before delivery, while 23.9% reported mild pain and 17.5% reported severe pain. Following delivery, 15.8% reported mild pain, 47.8% moderate pain, and 36.4% severe pain. Thus, 84.2% experienced moderate‐to‐severe pain after delivery. Overall, 88.2% of participants received medication for POP. Among all participants, 56.2% received painkillers, 34.4% received antibiotics, and 9.4% received pethidine. Because participants could receive more than one medication, these percentages are not mutually exclusive.

**TABLE 2:** Characteristics of pain and pain management factors among respondents.

Variable	Frequency	Percent (%)
*Did the pain start before delivery*		
No	181	60.9
Yes	116	39.1

*Nature of pain before delivery*		
No Pain	174	58.6
Mild pain	71	23.9
Severe pain	52	17.5

*Nature of pain after delivery*		
Mild pain	47	15.8
Moderate pain	142	47.8
Severe pain	108	36.4

*Received medication for post-delivery pain*		
No	35	11.8
Yes	262	88.2

*Taken medicine (after caesarean) name*		
Pain killer	167	56.2
Antibiotic	102	34.4
Pethidine	28	9.4

### 3.3. Patient Satisfaction and Treatment Outcomes

Maternal satisfaction served as a key indicator of postoperative care quality. Figure 1a presents the dichotomized satisfaction outcome used in the bivariate and multivariable analyses. Overall, 65% of mothers were classified as satisfied with their pain management, while 35% were classified as dissatisfied. Figure 1b presents the original four‐category distribution of the satisfaction item prior to dichotomization: 63% of respondents reported average satisfaction, 22% reported absolute satisfaction, and 9% reported being highly satisfied, while 6% reported no relief/dissatisfaction. Notably, despite this relatively high proportion reporting some degree of satisfaction, the majority of participants experienced considerable pain following surgery: 84.2% reported moderate‐to‐severe pain after delivery (Table 2) and 72.1% rated their worst pain as severe (Table 3). This discrepancy between high reported satisfaction and high pain intensity indicates that current pain management practices, while generally acceptable to patients, do not adequately control POP for the majority of women. Regarding treatment safety, 66% of participants experienced no adverse drug effects (Figure 1a), and the 34% who reported side effects described them as mild. Given that only 9.4% of participants received an opioid analgesic (pethidine) and many reported receiving no or minimal pharmacological pain relief, this low rate of adverse effects likely reflects limited analgesic exposure overall rather than good tolerability of adequately dosed treatment.

**TABLE 3:** Descriptive statistics of postoperative pain, interference, emotional impact, adverse drug effects, and treatment‐related outcomes assessed using the APS‐POQ‐R (*n* = 297).

Items				
Pain intensity measures, *n* (%)				
Variables	Not at all	Mild	Moderate	Severe
Least pain in last 24 h	9 (3.0)	15 (5.1)	257 (86.5)	16 (5.4)
Worst pain in last 24 h	1 (0.3)	3 (1.0)	79 (26.6)	214 (72.1)
Severe pain in last 24 h	3 (1.0)	10 (3.4)	86 (29.0)	198 (66.7)

**Interference with activities and emotional impact, *n* (%)**				
**Variables**	**Not at all**	**Mild**	**Moderate**	**Severe**

Activities in bed (turning, sitting up, repositioning)	38 (12.8)	7 (2.4)	160 (53.9)	92 (31.0)
Activities out of bed (walking, sitting in chair, standing)	48 (16.2)	23 (7.7)	115 (38.7)	111 (37.4)
Anxious with pain	24 (8.1)	6 (2.0)	64 (21.5)	203 (68.4)
Depressed with pain	47 (15.8)	6 (2.0)	87 (29.3)	157 (52.9)
Frightened with pain	16 (5.4)	5 (1.7)	44 (14.8)	232 (78.1)
Feeling helpless	29 (9.8)	8 (2.7)	97 (32.7)	163 (54.9)

**Adverse drug effects, *n* (%)**				
**Variables**	**Not at all**	**Mild**	**Moderate**	**Severe**

Nausea	242 (81.5)	3 (1.0)	42 (14.1)	10 (3.4)
Drowsiness	167 (56.2)	6 (2.0)	99 (33.3)	25 (8.4)
Itchiness	148 (49.8)	4 (1.3)	127 (42.8)	18 (6.1)
Dizziness	194 (65.3)	9 (3.0)	75 (25.3)	19 (6.4)

**Pain relief and satisfaction, *n* (%)**				
**Variables**	**Not at all**	**Mild**	**Moderate**	**Severe**

Pain relief in first 24 h	104 (35.0)	74 (24.9)	102 (34.3)	17 (5.7)
Satisfaction with pain treatment	13 (4.4)	4 (1.3)	222 (74.7)	58 (19.5)
Involvement in taking decision regarding pain treatment	11 (3.7)	1 (0.3)	225 (75.8)	60 (20.2)

### 3.4. Factors Associated With Pain Management Satisfaction

Table 4 illustrates the bivariate associations between pain management satisfaction and key sociodemographic and clinical variables. Age played a significant role in satisfaction levels (*p* = 0.019), with younger mothers (≤ 18 years) reporting a 57.1% satisfaction rate, while the 19–24 age group showed the highest overall satisfaction at 75.2%. BMI also showed a significant association (*p* = 0.037); underweight respondents reported the highest satisfaction (81.8%), whereas obese respondents reported the lowest (40.0%). The educational and economic background of the household also influenced perceptions of care. Interestingly, women whose husbands had no formal education reported the highest satisfaction rate (91.7%, *p* = 0.005). Regarding family finances, women from lower‐income households (≤ 20,000 BDT) expressed significantly higher satisfaction (74.5%) than those in the highest income bracket (*p* = 0.036). Mothers who did not experience pain prior to delivery reported higher satisfaction (69.1%) than those who did (*p* = 0.043). Postoperatively, those experiencing moderate pain reported a higher satisfaction rate (72.5%) compared to those with severe pain (59.3%) or mild pain (55.3%, *p* = 0.030). The strongest association was observed with the use of pain‐relieving medication. Mothers who utilized medication reported a 60.7% satisfaction rate, an association that was highly statistically significant (*p* ≤ 0.001). Furthermore, the type of medication influenced outcomes (*p* = 0.004); mothers who received antibiotics (75.5%) or pethidine (75.0%) reported higher satisfaction than those receiving standard painkillers alone (56.9%); as antibiotics are not analgesic agents, this pattern more plausibly reflects that antibiotic receipt served as a marker of more comprehensive postoperative treatment rather than a direct pain‐relieving effect.

**TABLE 4:** Association of pain management status among sociodemographic and pain related variables of respondents.

Characteristics	Pain management satisfaction		*χ* ^2^ value	*p* value
	No *n* (%)	Yes *n* (%)		
*Respondent’s age (in years)*				
≤ 18	18 (42.9)	24 (57.1)	9.10	0.019^∗^
19–24	26 (24.8)	79 (75.2)		
25–28	45 (44.1)	57 (55.9)		
≥ 30	15 (31.2)	33 (68.8)		

*Age at first birth (in years)*				
≤ 19	57 (37.7)	94 (62.3)	7.32^a^	0.042^∗^
20–24	26 (26.8)	71 (73.2)		
25–29	15 (37.5)	25 (62.5)		
≥ 30	6 (66.7)	3 (33.3)		

*Body mass index*				
Underweight	2 (18.2)	9 (81.8)	8.50^a^	0.037^∗^
Normal weight	46 (34.6)	87 (65.4)		
Overweight	41 (32.0)	87 (68.0)		
Obese	15 (60.0)	10 (40.0)		

*Respondent’s education level*				
No education	1 (16.7)	5 (83.3)	6.16^a^	0.104
Primary	19 (43.2)	25 (56.8)		
Secondary	46 (40.4)	68 (59.6)		
Higher	38 (28.6)	95 (71.4)		

*Husband’s education level*				
No education	2 (8.3)	22 (91.7)	12.90	0.005^∗^
Primary	15 (39.5)	23 (60.5)		
Secondary	19 (52.8)	17 (47.2)		
Higher	68 (34.2)	131 (65.8)		

*Husband’s occupation*				
Service holder	40 (35.7)	72 (64.3)	2.16	0.540
Businessman	43 (38.7)	68 (61.3)		
Farmer	4 (26.7)	11 (73.3)		
Day laborer	17 (28.8)	42 (71.2)		

*Monthly family income (in BDT)*				
≤ 20,000	26 (25.5)	76 (74.5)	6.67	0.036^∗^
20,001–25,000	13 (35.1)	24 (64.9)		
25,000+	65 (41.1)	93 (58.9)		

*Did the pain start before delivery*				
No	56 (30.9)	125 (69.1)	3.39	0.043^∗^
Yes	48 (41.4)	68 (58.6)		

*Nature of pain before delivery*				
No pain	56 (32.2)	118 (67.8)	3.07	0.216
Mild pain	31 (43.7)	40 (56.3)		
Severe pain	17 (32.7)	35 (67.3)		

*Nature of pain after delivery*				
Mild pain	21 (44.7)	26 (55.3)	7.04	0.030^∗^
Moderate pain	39 (27.5)	103 (72.5)		
Severe pain	44 (40.7)	64 (59.3)		

*Taken medicine to reduce the pain*				
No	1 (2.9)	34 (97.1)	18.03	≤ 0.001^∗∗^
Yes	103 (39.3)	159 (60.7)		

*Name of taken medicine*				
Pain killer	72 (43.1)	95 (56.9)	11.21	0.004^∗^
Antibiotic	25 (24.5)	77 (75.5)		
Pethidine	7 (25.0)	21 (75.0)		

^a^Likelihood ratio test.

^∗∗^Significant *p* < 0.001.

^∗^Significant *p* < 0.05.

### 3.5. Factors Associated With Pain Management Satisfaction

To identify factors independently associated with pain management satisfaction, binary logistic regression analysis was performed (Table 5). After adjustment for potential confounders, women aged 25–29 years had lower odds of satisfaction than those aged ≥ 30 years (AOR = 0.43, 95% CI: 0.19–0.96; *p* = 0.039). Women whose husbands had secondary education also had lower odds of satisfaction than those whose husbands had higher education (AOR = 0.32, 95% CI: 0.14–0.75; *p* = 0.009). Overweight women had higher odds of satisfaction than obese women (AOR = 3.27, 95% CI: 1.26–8.49; *p* = 0.014). Women who did not receive medication had higher odds of satisfaction than those who received medication (AOR = 5.49, 95% CI: 1.37–22.06; *p* = 0.016). The association between medication type and satisfaction should be interpreted cautiously because the reported confidence interval for painkillers includes 1.00 (AOR = 0.39, 95% CI: 0.14–1.10), despite the reported *p* value of 0.035; this discrepancy should be verified against the original regression output. The Hosmer–Lemeshow test indicated no evidence of poor model fit (*p* = 0.399), while the model showed good discriminatory ability (AUC = 0.769).

**TABLE 5:** Predictors of postoperative pain management satisfaction: crude and adjusted odds ratios.

Indicators	Unadjusted OR (95% CI)	*p* value	Adjusted OR (95% CI)	*p* value
*Respondent’s age*				
≤ 18	0.61 (0.26, 1.44)	0.256	0.48 (0.18, 1.31)	0.152
19–24	1.38 (0.65, 2.94)	0.401	1.04 (0.45, 2.41)	0.930
25–29	0.58 (0.28, 1.19)	0.035^∗^	0.43 (0.19, 0.96)	0.039^∗^
≥ 30	Reference		Reference	

*Husband’s education level*				
No education	5.66 (1.27, 25.18)	0.023^∗^	2.97 (0.55, 16.11)	0.208
Primary	0.77 (0.38, 1.57)	0.474	0.64 (0.27, 1.52)	0.391
Secondary	1.22 (0.69, 2.15)	0.035	0.32 (0.14, 0.75)	0.009^∗^
Higher	Reference		Reference	

*Monthly family income*				
≤ 20,000	2.04 (1.18, 3.53)	0.010^∗^	1.83 (0.84, 3.99)	0.130
20,001–25000	1.29 (0.62, 2.72)	0.503	1.01 (0.43, 2.41)	0.978
25,000+	Reference		Reference	

*BMI*				
Underweight	1.50 (0.46, 4.92)	0.503	1.57 (0.42, 5.85)	0.498
Normal	3.15 (1.30, 7.60)	0.011^∗^	2.49 (0.94, 6.67)	0.068
Overweight	3.43 (1.42, 8.33)	0.006^∗^	3.27 (1.26, 8.49)	0.014^∗^
Obese	Reference		Reference	

*Did the pain start before delivery*				
No	1.58 (0.97, 2.56)	0.067	1.61 (0.89, 2.91)	0.117
Yes	Reference		Reference	

*Nature of pain after delivery*				
Mild pain	0.85 (0.43, 1.69)	0.648	0.61 (0.25, 1.47)	0.269
Moderate pain	1.82 (1.07, 3.09)	0.028^∗^	1.70 (0.88, 3.26)	0.113
Severe pain	Reference		Reference	

*Taken medicine (pain killer) to reduce the pain after delivery*				
No	7.19 (2.15, 24.06)	0.001^∗^	5.49 (1.37, 22.06)	0.016^∗^
Yes	Reference		Reference	

*Name of taken medicine*				
Pain killer	0.44 (0.18, 1.09)	0.076	0.39 (0.14, 1.10)	0.035^∗^
Antibiotic	1.03 (0.39, 2.70)	0.957	0.55 (0.18, 1.65)	0.284
Pethidine	Reference		Reference	

^∗∗^Significant *p* < 0.001.

^∗^Significant *p* < 0.05.

### 3.6. Postoperative Pain Management Outcomes (APS‐POQ‐R)

Table 3 summarizes the multidimensional POP management outcomes assessed using the APS‐POQ‐R during the first 24 h after CD. Most participants (86.5%) rated their least pain as moderate, while 72.1% rated their worst pain as severe. Furthermore, 66.7% reported spending a severe proportion of the first 24 h in pain. Pain substantially interfered with physical activities, with moderate‐to‐severe interference reported for activities in bed by 84.9% of participants and for activities out of bed by 76.1%. Severe emotional impact was reported for fear (78.1%), anxiety (68.4%), helplessness (54.9%), and depression (52.9%). Adverse drug effects were generally less severe than pain‐related outcomes. Most participants reported no nausea (81.5%) or dizziness (65.3%), whereas moderate‐to‐severe drowsiness (41.7%) and itchiness (48.9%) were more frequently reported. Regarding pain relief, 35.0% reported no pain relief during the first 24 h, whereas only 5.7% reported severe/complete pain relief. Most participants (96.0%) reported at least moderate involvement in decisions regarding their pain treatment. However, only 22 participants (7.4%) reported receiving information about their pain‐treatment options, while 275 (92.6%) reported receiving no such information (Table S3). Among those who received information, the median perceived helpfulness score was 6.0 (range: 0–10).

### 3.7. Trends in Pain Severity and Management Satisfaction

The distribution of pain intensity, as illustrated in Figure S1, reveals a concerning trend where “least pain” reports cluster toward the middle of the scale, while “worst pain” and “severe pain” exhibit sharp upward trajectories, with the former peaking at over 55% in the highest intensity ranges (scores 8–10). This physical burden is further characterized by a “satisfaction and pain paradox” in Figure S2, which highlights a complex intersection of clinical outcomes: while satisfaction with pain relief reaches a peak of 38%, this milestone coincides with simultaneous spikes in severe pain reports (38%) and medication‐related side effects (29.5%). Furthermore, pain interference remains a persistent barrier to recovery, peaking at 18.9%, which collectively indicates that current management strategies frequently fall short of an optimal balance where high‐quality relief is achieved without the compromise of severe pain recurrence or increased side effects.

## 4. Discussion

This study examined POP severity and patient satisfaction among 297 women following CD in Rajshahi District, Bangladesh, using the multidimensional APS‐POQ‐R. Three main findings emerged. First, despite substantial pain intensity and functional and emotional burden in the first 24 h postoperatively, 65% of women nonetheless reported satisfaction with their pain management. Second, maternal age, BMI, and receipt of pharmacological analgesia were independently associated with satisfaction in multivariable analysis. Third, very few women (7.4%) reported receiving information about their pain‐treatment options, despite 96.0% reporting moderate‐to‐high involvement in treatment decisions. This discordance is suggestive of gaps in patient‐provider communication rather than in the volume of care delivered. These findings are discussed below in relation to existing literature.

Women aged 25–29 years were significantly less likely to report dissatisfaction than those aged ≥ 30 years (AOR = 0.43; 95% CI: 0.19–0.96) [45]. BMI emerged as a robust physical determinant of dissatisfaction: overweight women were over three times more likely to report poor satisfaction than obese women, the reference category (AOR = 3.27; 95% CI: 1.26–8.49). This is consistent with evidence that adipose tissue alters the pharmacokinetics of standard analgesic dosing, potentially resulting in suboptimal pain relief among women with higher BMI [46, 47]. Socioeconomic factors contributed less consistently to satisfaction. Women whose husbands had secondary‐level education reported lower satisfaction than those with higher‐educated spouses (AOR = 0.32; 95% CI: 0.14–0.75) [48, 49]. Although lower household income was associated with higher satisfaction in bivariate analysis (74.5% among the lowest income group vs. lower rates in higher‐income groups), this association did not persist after adjustment, suggesting that clinical and treatment‐related factors, rather than household income alone, more directly shaped women’s pain experiences in this cohort [50]. The strongest determinant of dissatisfaction was the absence of pharmacological pain relief: women who did not receive pain‐relieving medication were 5.49 times more likely to report dissatisfaction (95% CI: 1.37–22.06) [51]. Given that analgesics are part of standard postoperative protocol at all study facilities, this large effect size more plausibly reflects a gap in patient‐provider communication than a true absence of treatment. A small subgroup (*n* = 35) who reported not receiving medication showed markedly lower satisfaction (2.9%) than those who did (60.7%), a difference that may partly reflect either genuine undertreatment or a failure to recognize medication received as pain‐specific care [52]. Women receiving standard nonopioid painkillers alone also showed lower adjusted odds of satisfaction relative to those receiving pethidine (AOR = 0.39; 95% CI: 0.14–1.10) [53, 54]. It should be noted that medication data in this study were self‐reported and categorized broadly (painkiller, antibiotic, and pethidine), without information on specific agents, dosages, timing, or intraoperative anesthetic technique; these findings should therefore be interpreted as indicative of limited analgesic exposure overall, rather than a precise characterization of prescribing adequacy [55]. Notably, in bivariate analysis, women who received antibiotics (75.5%) or pethidine (75.0%) reported higher satisfaction than those receiving painkillers alone (56.9%); because antibiotics are not analgesic agents, this pattern more plausibly reflects that antibiotic receipt served as a marker of more comprehensive postoperative treatment overall, rather than indicating a direct pain‐relieving effect. Pain severity in this cohort was substantial by any categorical threshold: 72.1% of women rated their worst pain as severe and 66.7% reported spending a severe proportion of the 24‐h period in pain [56]. This intensity translated directly into functional impairment, with moderate‐to‐severe interference reported for activities in bed (84.9%) and out of bed (76.1%), consistent with prior evidence that inadequate pain control delays physical recovery and complicates early maternal–infant interaction [57]. Emotional distress was similarly pronounced, with severe levels reported for fright (78.1%), anxiety (68.4%), helplessness (54.9%), and depression (52.9%). Fright and anxiety were more frequently reported at severe intensity than depression in this cohort, indicating that the acute emotional response to POP in the immediate 24‐h period may be dominated by fear and anxiety rather than depressive symptoms, which may emerge or predominate over a longer recovery trajectory [58, 59]. An important paradox emerged between reported satisfaction and objective pain burden: 65% of women reported satisfaction despite 84.2% reporting moderate‐to‐severe pain after delivery (Table 2) and despite 35.0% reporting no pain relief at all in the first 24 h. A comparable pattern has been described by Schwenkglenks et al. [60] in the international PAIN OUT registry, using an instrument closely related to the APS‐POQ‐R to examine correlates of satisfaction with POP treatment across surgical patients. That study found that three items accounted for the majority of variance in satisfaction: the extent of pain relief obtained, the extent to which patients were allowed to participate in decisions about their pain management, and whether patients wished to have received more treatment for their pain [61]. Our findings are broadly consistent with the first two of these correlates: pain relief and involvement in treatment decisions were both linked to satisfaction in the present cohort, with 96.0% of women reporting moderate‐to‐high involvement in decision‐making. However, we were unable to examine the third correlate, patients’ wish for additional treatment, as this item is not included in the version of the APS‐POQ‐R administered in this study, representing a notable limitation relative to the PAIN OUT registry findings and points to a direction for future instrument adaptation in this setting. This satisfaction–pain discordance may also be partly explained by the striking gap between involvement and information: although 96.0% of women reported being involved in treatment decisions, only 7.4% reported having received any information about their pain‐treatment options (Table 3). Among the small subset who did receive information, perceived usefulness was only moderate (median helpfulness score 6.0, range 0–10). This suggests that women’s sense of ‘involvement’ may not have been grounded in meaningful, informed decision‐making. Instead, satisfaction ratings in this setting may be shaped largely by relational aspects of care attentiveness, responsiveness, and perceived inclusion rather than by the adequacy of pain control actually achieved or the substance of information provided [28]. Cultural norms in South Asian settings, where postoperative discomfort may be perceived as an expected consequence of childbirth, could further contribute to women rating moderate relief as an acceptable outcome even amid substantial unrelieved pain [26]. Reported adverse drug effects were comparatively mild: most women reported no nausea (81.5%) or dizziness (65.3%), although moderate‐to‐severe drowsiness (41.7%) and itchiness (48.9%) were relatively common. This should not be interpreted as evidence that current analgesic dosing is adequate and well tolerated. Given that only 9.4% of women received an opioid analgesic (pethidine) and pain and interference levels remained high throughout the cohort, the low rate of more serious adverse effects (nausea and dizziness) more plausibly reflects limited analgesic exposure than genuine tolerability of adequately dosed treatment. Taken together, the high pain intensity, substantial functional and emotional burden, and comparatively low rate of adverse effects observed in this cohort suggest that current postoperative analgesic practices in this setting may be conservative, potentially under‐treating pain rather than optimizing comfort within a safe therapeutic range. However, because information on intraoperative anesthesia type and intraoperative analgesic use was not collected, we cannot determine the extent to which these pain outcomes reflect gaps in postoperative systemic analgesia specifically, versus anesthetic technique, or both; this distinction should be clarified in future research incorporating anesthesia records.

### 4.1. Strength and Limitations of the Study

This study provides robust, multidimensional evidence on POP management among 297 mothers in Rajshahi, capturing demographic, socioeconomic, and psychological recovery factors across a multicenter sample spanning government, private, and specialized‐clinic facilities. This design reflects real‐world clinical variation, though it also carries several limitations. The cross‐sectional design limits causal inference, and self‐reported data are susceptible to recall bias. Restricting assessment to the first 24 postoperative hours precludes evaluation of longer‐term pain trajectories, and findings are specific to Rajshahi, which may limit generalizability. Although recruitment spanned multiple facility types, differences in pain management practices and outcomes between public and private settings were not formally compared; future studies should directly examine this. Several clinically relevant variables were not captured. Most notably, information on intraoperative anesthesia type (spinal, epidural, or general) and intraoperative analgesic use (e.g., neuraxial opioids and local anesthetic techniques) was not collected, as data collection relied on participants’ postoperative self‐report rather than anesthesia or clinical chart review. This is a substantive limitation: anesthetic technique is a major determinant of early POP trajectories, and its absence limits our ability to determine whether the high pain scores observed in this cohort reflect gaps in postoperative systemic analgesia, intraoperative anesthetic technique, or both. Similarly, although approximately 39% of participants reported pre‐delivery pain, a factor significantly associated with postoperative satisfaction, its specific source (e.g., labor contractions versus other causes) was not characterized. Medication use was also categorized broadly (painkiller, antibiotic, and pethidine) based on self‐report, without data on specific agents, dosages, or administration schedules, limiting conclusions about analgesic adequacy or timing; consequently, our findings can indicate that POP management appeared limited in this cohort (e.g., low opioid use and substantial unrelieved pain) but cannot fully characterize the extent or structure of analgesic care actually provided. Future studies incorporating intraoperative anesthetic records and clinical chart review would allow more precise characterization of these factors in relation to pain and satisfaction outcomes. In addition, satisfaction with pain management is itself a complex, multifactorial construct that may be shaped by relational and contextual dimensions of care such as attentiveness, communication, and patient expectations rather than by pain control alone. This may partly explain the discordance observed between high reported satisfaction and substantial unrelieved pain in this cohort, and it suggests that satisfaction, while a valuable patient‐centered indicator, should not be interpreted as a direct proxy for the clinical adequacy of pain management. Finally, variables such as pre‐existing mental health conditions, cultural attitudes toward pain, and adherence to clinical protocols were not assessed, leaving potential for unmeasured confounding.

## 5. Conclusions

This study demonstrates an urgent need to improve POP management for women undergoing CD in Rajshahi District. While current practices provide a baseline of safety, they frequently fall short of adequate clinical and psychological care. A substantial “satisfaction–pain paradox” was observed: although 65% of women reported satisfaction with pain management, this occurred alongside high pain intensity, with 84.2% reporting moderate‐to‐severe pain and over one‐third reporting no pain relief at all in the first 24 h. The impact of inadequate pain control extended beyond physical limitations, manifesting in substantial emotional distress, most prominently fright and anxiety, alongside helplessness and depression. Maternal age, BMI, and receipt of pharmacological analgesia emerged as the primary determinants of satisfaction, with overweight women and those receiving no pain‐relieving medication at particular risk of dissatisfaction. Notably, despite high reported involvement in treatment decisions, very few women had received any information about their pain‐treatment options, pointing to a gap in patient‐provider communication that may partly explain the observed satisfaction–pain discordance. This pattern is consistent with prior evidence that the extent of pain relief and involvement in care decisions are key correlates of postoperative satisfaction, underscoring the value of assessing patients’ unmet treatment needs more directly in future research. Given the absence of a documented, standardized pain management protocol across facilities and the low observed use of stronger analgesics despite high pain intensity, these findings suggest a need for future research to formally evaluate and, where indicated, move toward more individualized, multimodal analgesic strategies. Priorities should include earlier and more systematic pain assessment, tailoring analgesic approaches to individual physical profiles such as BMI, and strengthening communication with patients about the purpose, timing, and expected effects of pain treatment. Addressing both the physical and emotional dimensions of POP is likely to improve maternal recovery, functional outcomes, and early maternal–infant bonding among women undergoing CD in this setting. Translating these findings into practice will require coordinated commitment from clinicians, facility administrators, and policymakers; while implementing such change may be challenging, improved pain management stands to benefit maternal recovery, infant well‐being, and the broader quality of obstetric care in this region.

## Author Contributions

Conceptualization: Farhana Hasan.

Data curation: Abu Sayed Md. Al Mamun and Mst Sabina Yeasmin Swapna.

Formal analysis: Farhana Hasan, Abu Sayed Md. Al Mamun, Md. Kaderi Kibria, and Md. Golam Hossain.

Investigation: Md. Kaderi Kibria.

Methodology: Farhana Hasan, Abu Sayed Md. Al Mamun, and Md. Golam Hossain.

Resources: Md. Kaderi Kibria, Mst Sabina Yeasmin Swapna, and Dr. Masuma Amanullah.

Supervision: Md. Golam Hossain.

Validation: Abu Sayed Md. Al Mamun.

Writing–original draft: Farhana Hasan.

Writing–review and editing: Abu Sayed Md. Al Mamun, Md. Kaderi Kibria, and Md. Golam Hossain.

## Funding

No funding was received for this manuscript.

## Conflicts of Interest

The authors declare no conflicts of interest.

## Supporting Information

Additional supporting information can be found online in the Supporting Information section.

## Supporting information


**Supporting Information** Table S1 presents the distribution of study participants by healthcare facility (*n* = 297). Table S2 presents the item–total statistics of the APS‐POQ‐R scale, including item–total correlations and reliability coefficients, to assess the internal consistency of the instrument used in this study. Table S3 presents APS‐POQ‐R information received and nonpharmacological pain management elements. Figure S1 illustrates the distribution and trends in POP intensity among respondents during the first 24 h following CD. Figure S2 demonstrates the relationships among pain management satisfaction, pain intensity, and reported treatment side effects among post‐caesarean women.

## Data Availability

The data that support the findings of this study are available from the corresponding author upon reasonable request.
