# Supplementary material for: Factors Associated With Postoperative Pain Severity and Patient Satisfaction Among Post‐Caesarean Women in Rajshahi District, Bangladesh
Source: Anesthesiol Res Pract. 2026 Sep 18;2026:1056316. doi: 10.1155/anrp/1056316 (PMC13586787; doi:10.1155/anrp/1056316)
Supplement: Supplementary file 1 [file ANRP-2026-1056316-s001.docx]

Supplementary File

**Table S1:** Distribution of study participants by healthcare facility (*n* = 297)

| SN | Hospital Names | Hospital Types | Participants |
| --- | --- | --- | --- |
| 1. | Rajshahi Medical College Hospital | Government | 93 |
| 2. | Amana Hospital Ltd. | Private | 48 |
| 3. | Zamzam Islami Hospital | Private | 48 |
| 4. | Rajshahi Royel Hospital Private Limited | Private | 48 |
| 5. | Uttora Clinic | Clinic | 20 |
| 6. | Padma Clinic | Clinic | 20 |
| 7. | Dolphin Clinic | Clinic | 20 |

**Table S2:** Item-total statistics of the APS-POQ-R scale

| **Items** | **Corrected item-total correlation** | **Statement** |
| --- | --- | --- |
| LP (Least pain in 24 h) | 0.72 | Valid |
| WP (worst pain in 24 h) | 0.77 | Valid |
| SP (Severe pain in 24 h %) | 0.74 | Valid |
| AB (Pain interfered activities in bed) | 0.76 | Valid |
| AO (Pain interfered activities out of bed) | 0.74 | Valid |
| FA (Pain interfered with falling asleep) | 0.43 | Valid |
| SA (Pain interfered staying asleep) | 0.41 | Valid |
| ANX (anxiety caused by pain) | 0.61 | Valid |
| DEP (Depression caused by pain) | 0.51 | Valid |
| FRI (Fright caused by pain) | 0.48 | Valid |
| HL (Helpless caused by pain) | 0.50 | Valid |
| PR (Pain relief %) | 0.41 | Valid |
| DM (Decision regarding pain treatment) | 0.39 | Valid |
| SPT (Pain treatment satisfaction) | 0.37 | Valid |

**Table S3: APS-POQ-R** information received and non-pharmacological pain management elements

| **Variables** | **Frequency** | **Percentage** |
| --- | --- | --- |
| **Information about pain treatment management** | | |
| Received | 22 | 7.4 |
| Not received | 275 | 92.6 |
| **Helpfulness of information about pain management treatment (receved=22)** | | |
| Median (Min-Max) | 6.0 (0.0-10.0) | |

**Note:** Numerical data are not normally distributed, and thus presented with a median value and range. Categorical data is presented with the amount and percentage.

**Figure S1:** Distribution and trends of postoperative pain intensity among respondents within 24 hours.

**Figure S2:** The relationship between pain management satisfaction, pain Intensity and treatment side effects.
